# Mentalizing impairments and hypermentalizing bias in individuals with first-episode schizophrenia-spectrum disorder and at-risk mental state: the differential roles of neurocognition and social anxiety

**DOI:** 10.1007/s00406-024-01830-y

**Published:** 2024-07-03

**Authors:** Harry Kam Hung Tsui, Yingqi Liao, Janet Hsiao, Yi Nam Suen, Eric Wai Ching Yan, Lap-Tak Poon, Man Wah Siu, Christy Lai Ming Hui, Wing Chung Chang, Edwin Ho Ming Lee, Eric Yu Hai Chen, Sherry Kit Wa Chan

**Affiliations:** 1 Department of Psychiatry, Li Ka Shing Faculty of Medicine, The University of Hong Kong, Pok Fu Lam, Hong Kong SAR; 2 Division of Social Science, Hong Kong University of Science & Technology, Clear Water Bay, Hong Kong SAR, China; 3 Kowloon Hospital, Mong Kok, Hong Kong SAR; 4 Department of Psychiatry, United Christian Hospital, Kwun Tong, Hong Kong SAR; 5 Department of Psychiatry, Kwai Chung Hospital, Kwai Chung, Hong Kong SAR; 6 The State Key Laboratory of Brain and Cognitive Sciences, The University of Hong Kong, Pok Fu Lam, Hong Kong SAR

**Keywords:** At-risk mental states, First-episode schizophrenia, Theory of mind, Social cognition, Neurocognition, Social anxiety

## Abstract

Mentalizing, or theory of mind (ToM), impairments and self-referential hypermentalizing bias are well-evident in schizophrenia. However, findings compared to individuals with at-risk mental states (ARMS) are inconsistent, and investigations into the relationship between social cognitive impairments and social anxiety in the two populations are scarce. This study aimed to examine and compare these deficits in first-episode schizophrenia-spectrum disorder (FES) and ARMS, and to explore potential specific associations with neurocognition and symptomatology. Forty patients with FES, 40 individuals with ARMS, and 40 healthy controls (HC) completed clinical assessments, a battery of neurocognitive tasks, and three social cognitive tasks. The comic strip and hinting tasks were used to measure non-verbal and verbal mentalizing abilities, and the gaze perception task was employed to assess self-referential hypermentalizing bias. FES and ARMS showed comparable mentalizing impairments and self-referential hypermentalizing bias compared to HC. However, only ambiguous self-referential gaze perception (SRGP) bias remained significantly different between three groups after controlling for covariates. Findings suggested that self-referential hypermentalizing bias could be a specific deficit and may be considered a potential behavioral indicator in early-stage and prodromal psychosis. Moreover, working memory and social anxiety were related to the social cognitive impairments in ARMS, whereas higher-order executive functions and positive symptoms were associated with the impairments in FES. The current study indicates the presence of stage-specific mechanisms of mentalizing impairments and self-referential hypermentalizing bias, providing insights into the importance of personalized interventions to improve specific neurocognitive domains, social cognition, and clinical outcomes for FES and ARMS.

**Supplementary Information:**

The online version contains supplementary material available at 10.1007/s00406-024-01830-y.

## Introduction

Social cognition is the perception, processing, and interpretation of social stimuli, such as facial expressions, gaze direction, and verbal communication, to effectively communicate with others and adapt to the social world [1]. Mentalizing ability, or theory of mind (ToM), as one of the major components of social cognition, generally refers to the ability to appreciate others’ thoughts, intentions, and emotions [2]. Significant social cognitive impairments have been regarded as core features in patients with schizophrenia-spectrum disorder [3], even during the first episode of the illness [4], which are significantly associated with poor psychosocial functioning [5]. Earlier meta-analyses [6–8] have suggested that individuals with at-risk mental state (ARMS), who exhibit attenuated psychotic symptoms and an increased risk of developing a psychotic disorder, generally have moderate mentalizing impairments compared to healthy controls (HC) and are less severe than patients with first-episode psychosis. However, the results of recent studies are inconsistent, possibly due to various factors, including the multidimensional nature of mentalizing ability, the use of different mentalizing tasks, and clinical variations among samples. Some suggested that ARMS individuals had an intact mentalizing ability as HC [9], whereas others reported that ARMS individuals had similar mentalizing impairments as patients with schizophrenia [10]. Furthermore, previous studies comparing individuals with first-episode schizophrenia (FES) and ARMS usually relied on a single mentalizing task [8]. This could have limited the scope of mentalizing ability assessed in the samples, that should include verbal and non-verbal comprehensions, cognitive and affective mentalizing abilities [11]. Moreover, the differential associations between the social cognitive task designs with symptoms and neurocognition added complexity to the inconsistent findings [12, 13]. A study by Green et al. [14] has suggested that ARMS and FES exhibit different patterns of association between social cognitive impairments and symptomatology, indicating that the relationship may be influenced by the specific stages or distinct symptom presentations.

Social cognition is considered as one of the major domains of neurocognition. While deficits in general neurocognitive functions are posited to contribute to mentalizing impairments, the nature and extent of this contribution remained unclear [12]. Studies have indeed suggested that a wide range of neurocognitive function domains were significantly related to mentalizing impairments in patients with FES [15], especially executive functions [16, 17]. On the other hand, findings on the associations between neurocognition and social cognition in the ARMS population were scarce and less definitive [8]. Few studies have reported that working memory, processing speed [18, 19], executive functions [20, 21], and multiple neurocognitive domains [22] may be associated with the mentalizing impairments in ARMS. While some studies suggested that mentalizing impairments in schizophrenia and ARMS could be explained primarily by neurocognition [23], others did not support such findings [24]. Crucially, the patterns of relationship between neurocognition and social cognition were found to be different between ARMS and HC [18–20], which might be attributed to the unique psychopathological and cognitive deficits present in ARMS individuals. Existing research has not yet fully compared the ARMS and FES populations in this regard. It is thus possible that ARMS and FES populations display distinct patterns of association between neurocognition and mentalizing abilities.

Conceptually mentalizing errors could be understood in two directions: an impoverished or lack of mentalizing ability (hypomentalizing) and an excessive inference beyond typical social understanding (hypermentalizing). It has been proposed that negative and positive symptoms of schizophrenia are related to hypomentalizing and hypermentalizing errors respectively [25–27]. Particularly, few studies have indicated that patients with schizophrenia tended to exhibit a self-referential judgement in non-verbal social cues compared to HC, including gaze [25] and gesture perceptions [28], while such misattribution tendency would directly lead to misunderstandings and difficulties in social interactions. However, self-referential hypermentalizing tendency in the ARMS population has not been investigated thus far. Additionally, social anxiety has been proposed to negatively impact social cognitive functions in the general population and people with anxiety disorders [29, 30]. Despite the high prevalence of social anxiety in FES and ARMS populations [31, 32], its effects on the mentalizing abilities of these populations remained unexplored. Only one recent study by Williams et al. [33] found an association between social anxiety and social cognitive biases in CHR, where those with increased levels of social anxiety tended to mislabel neutral expressions as anger. Exploring the possible distinct relationships between mentalizing ability and self-referential bias with neurocognition and social anxiety in FES and ARMS individuals could offer valuable insight into understanding the psychopathological mechanisms of mentalizing impairments in individuals at different stages of psychosis and potentially inform future development of interventional approaches.

In the current study, we aimed to examine the mentalizing impairments and self-referential hypermentalizing tendency in FES and ARMS using three social cognitive tasks. We hypothesized that FES patients would have more mentalizing deficits and hypermentalizing errors than ARMS individuals, and that ARMS individuals would demonstrate these impairments compared to HC. Differential relationships between social cognitive performances with neurocognition and symptomatology in each population were studied. Social anxiety and neurocognition were hypothesized to be significantly associated with mentalizing impairments and self-referential hypermentalizing tendency in FES and ARMS. The findings of this study will allow better characterization of social cognitive impairments and understanding of their potential mechanisms among individuals with FES and ARMS.

## Material and methods

### Participants and procedures

A total of 120 participants, with 40 participants in each group matched with age and gender, were recruited in three groups: patients with first-episode schizophrenia-spectrum disorder (FES), individuals with at-risk mental states (ARMS), and healthy controls (HC). Participants with FES and ARMS were recruited from the outpatient unit of the Early Intervention Service for psychosis (EASY) programme in Hong Kong [34]. All diagnosis of schizophrenia-spectrum disorder was determined by trained psychiatrists based on the DSM-V criteria [35]. Help-seeking individuals were identified as ARMS by psychiatrists using the Comprehensive Assessment of At-Risk Mental States (CAARMS) [36], characterizing those with attenuated psychotic symptoms but did not fulfill any diagnoses of schizophrenia-spectrum disorder. Healthy individuals without a history of psychiatric disorders, nor a family history of psychiatric disorders were recruited from the community. Participants were excluded from all three groups based on the following criteria: (1) the presence of pervasive developmental disorders; (2) other major psychiatric disorders, such as mood and anxiety spectrum disorders; (3) a history of substance use or neurological disorders; (4) any auditory, speech, or visual impairments; and (5) moderate to severe learning disability. Clinicians identified eligible FES and ARMS individuals as meeting these criteria prior to their enrollment in the EASY program, with verification from medical records [34]. Healthy controls were screened based on self-report and past education history. Written informed consent was obtained from the participants and parents of those under 18. The authors assert that all procedures contributing to this work comply with the ethical standards of the relevant national and institutional committees on human experimentation and with the Helsinki Declaration of 1975, as revised in 2008. All procedures involving human participants were approved by the Institutional Review Board of the University of Hong Kong and the Hospital Authority Hong Kong West Cluster (IRB reference number: UW 13–205). Face-to-face interviews were conducted by a team of research clinicians and trained research assistants to assess participants’ symptomatology, neurocognition, and social cognitive performances. Clinical ratings were rated by research clinicians independently who were not involved in any treatment procedures for patients. All researchers received comprehensive training to strictly adhere to the standardized assessment protocols to minimize bias.

### Clinical assessments

Demographic variables, including age, gender, and years of education were obtained. Participants were assessed by the Peters et al. Delusion Inventory (PDI) for delusional ideation [37], the Ideas of Reference Interview Schedule (IRIS) for idea of reference [38], and the Liebowitz Social Anxiety Scale (LSAS) for social anxiety [39]. Only FES and ARMS participants were assessed for clinical symptoms using the Positive and Negative Syndrome Scale (PANSS) [40]. The Interview for Retrospective Assessment of Onset of Schizophrenia (IRAOS) was used to assess the duration of illness (DUI) and duration of untreated psychosis (DUP) of FES patients [41]. The Defined daily dose (DDD) of antipsychotics was calculated as the average maintenance dose prescribed per day to FES and ARMS participants at the time of assessment [42].

### Neurocognition assessments

Cognitive tasks were mainly extracted from the Wechsler Adult Intelligence Scale-Revised (WAIS-R) [43]. Neurocognition was classified into three neurocognitive domains as executive functions, processing speed, and working memory. The executive functions domain included the time difference between Trail Making Test (TMT) Part B and Part A of the test (B-A) assessing cognitive flexibility [44], as well as the arithmetic test measuring quantitative reasoning and problem-solving ability. TMT Part A and the digit symbol substitution test were used to measure processing speed. Digit span and letter-number-sequencing tests were implemented to assess working memory.

TMT consists of two parts: part A requires the participant to connect consecutively numbered circles as quickly as possible; Part B requires connecting numbered and lettered circles in alternating sequence. Scoring is based on the time taken to complete each part, with longer times indicating poorer performance. The arithmetic test requires participants to solve a series of oral arithmetic problems without the use of pencil and paper, and scores based on the number of correct responses. The digit symbol substitution test requires participants to match symbols with corresponding numbers using a key within a limited time, where scores are based on the number of correct matches. The digit span test consists of two components: the digit span forward and the digit span backward, where participants have to repeat numbers in the same order and reverse order respectively. Scoring is based on the longest string of numbers correctly repeated. Letter-number-sequencing test requires participants to hear a mixed series of numbers and letters and must recall the numbers in ascending order and letters in alphabetical order. The score is the total number of correctly sequenced trials. These tests have been validated and have shown good psychometric properties [43, 44].

### Assessments of social cognitive function

The comic strip task was used to examine non-verbal mentalizing ability [45, 46]. Participants were asked to infer the character’s mental state and subsequent behavior in 28 social scenarios. In each scenario, three pictures were shown in sequential order simultaneously, and participants were instructed to choose the most probable ending of the scenario with multiple choices of another three pictures. The hinting task was employed to assess verbal mentalizing ability [47]. The task had 10 interpersonal communication scenarios, and participants were asked to infer the mental states or thoughts of the characters in each scenario by one or two open-ended questions. A score of 0, 1, or 2 was given based on the correctness of their inferences and the need for hints. A more standardized and stringent scoring method proposed by Klein et al. [48] was implemented to improve the psychometric properties of the hinting task. The ceiling effects of the social cognitive tasks were determined by obtaining perfect scores in the hinting task (n = 12/120; 10%) and comic strip task (n = 5/120; 4.2%) [49]. The skewness statistics of the hinting task and comic strip task were − 0.868 and − 0.513 respectively, which fall within the excellent range of − 1 to + 1 [50]. The internal consistency (Cronbach’s alpha) of comic strip task in the current samples was 0.86 in FES, 0.82 in ARMS, and 0.70 in HC, whereas the internal consistency of hinting task was 0.78 in FES, 0.79 in ARMS, and 0.66 in HC.

The gaze perception task was a computerized task measuring self-referential gaze perception (SRGP) which indicated participants’ hypermentalizing tendency [25, 51]. The task comprised six blocks with 30 trials for each block, and each trial consisted of a stimulus showing a neutral face with varying gaze direction from 0° to 30°, each presented for 200 ms. Participants were asked to respond to the question “Do you feel as if the person in the picture is looking at you?” after each stimulus by clicking on a specified mouse key (Supplementary Fig. 1). Stimuli with different gaze directions were categorized into center (0°, 5°), ambiguous (10°, 15°), and unambiguous (20°, 25°, 30°) gazes. SRGP rates of ambiguous and unambiguous conditions were calculated as the proportion of perceiving averted gazes as self-referential. A higher SRGP rate represents a higher self-referential hypermentalizing bias. The internal consistency of SRGP rates was 0.94 in FES, 0.92 in ARMS, and 0.72 in HC.

### Statistical analysis

A five-factor PANSS model was utilized, including positive symptoms, negative symptoms, disorganization, depression-anxiety, and excitement/activity [52]. Cognitive tasks were standardized as z-scores and averaged into the three neurocognitive domains. The average of all neurocognitive domains was calculated to derive a cognitive composite score. One-way ANOVAs were conducted to compare the demographics, clinical characteristics, neurocognition, and social cognitive performances between the FES, ARMS, and control groups. Kruskal–Wallis H Test and Mann–Whitney U test were used for PANSS scores and SRGP rates due to their non-parametric nature. Bonferroni pairwise post hoc tests were performed to correct for multiple testing and examine group differences. The non-parametric data were transformed using the Box-Cox transformation for later covariate analyses [53]. ANCOVA was used to explore the social cognitive deficits between groups while controlling for age, gender, years of education, and general neurocognition. Pearson’s correlational analyses were conducted to evaluate the associations between social cognitive performances with clinical characteristics and neurocognitive domains in different groups. Correlational analysis of the full sample was also performed to examine the symptom continuum across clinical categories with the multiple testing corrected with Benjamini–Hochberg procedure. Significant covariates were then selected for regression analyses, accounting for demographics to investigate the importance of the significant variables. All statistical analyses were performed using SPSS version 28.0.

## Results

### Demographic and clinical characteristics of the samples

The demographics, clinical measurements, and behavioral performances of all participants were summarized and compared in Table 1. The mean age was 24.60 (S.D. = 6.28), 23.78 (S.D. = 7.95) and 25.25 (S.D. = 7.53) in FES, ARMS, and HC respectively. No significant difference was found in age and gender between the three groups. However, HC had significantly higher educational attainment than FES (*p* < 0.001) and ARMS participants (*p* < 0.001). FES patients had a significantly higher PANSS score than ARMS individuals (*U* = 12.622, *p* < 0.001) (Table 1). Significant three group differences were observed in IRIS item score (*F* = 44.458, *p* < 0.001), LSAS total score (*F* = 14.219, *p* < 0.001), and PDI total score (*F* = 22.358, *p* < 0.001) with ARMS and FES groups demonstrating significantly higher scores than HC (Table 1). FES patients had a higher IRIS item score than ARMS individuals (*p* = 0.005), but there were no differences between FES and ARMS groups in PDI and LSAS total scores.

**Table 1:** Comparison of the three groups on demographics, clinical characteristics, cognitive and social cognitive functions

Variables	FES (*N* = 40)	ARMS (*N* = 40)	HC (*N* = 40)	*F/χ*^*2*^/*U*	*p* value	Pairwise comparison
						Group comparisons
Demographics						
Age (mean, S.D.)	24.60 (6.28)	23.78 (7.95)	25.25 (7.53)	0.412	0.664	
Gender (male, n%)	19 (47.5%)	16 (40%)	19 (47.5%)	0.606	0.739	
Years of education (mean, S.D.)	13.05 (2.98)	12.60 (2.65)	15.10 (2.44)	9.759	** < 0.001**	HC > ARMS***, HC > FES***
Defined daily dose (mean, S.D.)	0.95 (0.89)	0.28 (0.48)	N/A	21.158	** < 0.001**	
Medicated with antipsychotics (n%)	38 (95%)	21 (47.5%)	N/A	N/A	N/A	
Duration of illness, days (mean, S.D.)	302.85 (436.45)	N/A	N/A	N/A	N/A	
Duration untreated psychosis, days (mean, S.D.)	208.55 (374.97)	N/A	N/A	N/A	N/A	
Clinical characteristics						
PANSS positive (mean, S.D.)	17.33 (3.62)	10.95 (4.06)	N/A	33.183	** < 0.001**	
PANSS negative (mean, S.D.)	13.18 (5.27)	12.68 (5.85)	N/A	0.283	0.594	
PANSS disorganization (mean, S.D.)	10.78 (3.30)	9.55 (2.63)	N/A	4.322	**0.038**	
PANSS depression-anxiety (mean, S.D.)	10.18 (3.32)	11.00 (3.98)	N/A	0.908	0.341	
PANSS excitement/activity (mean, S.D.)	5.10 (2.04)	4.53 (1.13)	N/A	1.421	0.233	
IRIS item (mean, S.D.)	3.93 (2.58)	2.60 (1.78)	0.14 (0.41)	44.458	** < 0.001**	FES > ARMS**, FES > HC***, ARMS > HC***
LSAS total (mean, S.D.)	51.21 (31.63)	59.93 (30.38)	28.70 (16.49)	14.219	** < 0.001**	FES > HC***, ARMS > HC***
PDI total (mean, S.D.)	73.92 (55.81)	68.64 (49.82)	14.40 (14.81)	22.358	** < 0.001**	FES > HC***, ARMS > HC***
Neurocognition						
Executive functions scores (mean, S.D.)	− 1.93 (1.48)	− 1.39 (1.32)	0.00 (0.78)	26.401	** < 0.001**	HC > FES***, HC > ARMS***
Processing speed scores (mean, S.D.)	− 2.32 (1.69	− 1.92 (2.16)	0.00 (0.79)	25.212	** < 0.001**	HC > FES***, HC > ARMS***
Working memory socres (mean, S.D.)	− 1.66 (1.02)	− 1.42 (1.43)	0.00 (0.86)	22.697	** < 0.001**	HC > FES***, HC > ARMS***
Cognitive composite scores (mean, S.D.)	− 1.97 (0.99)	− 1.57 (1.35)	0.00 (0.58)	41.553	** < 0.001**	HC > FES***, HC > ARMS***
Social cognition						
Comic strip task (mean, S.D.)	21.28 (4.17)	20.54 (5.97)	25.03 (2.48)	11.752	** < 0.001**	HC > FES***, HC > ARMS***
Hinting task (mean, S.D.)	16.23 (2.52)	16.50 (3.27)	17.73 (1.40)	4.028	**0.020**	HC > FES*, HC > ARMS^
Central SRGP rate (mean, S.D.)	0.76 (0.14)	0.81 (0.16)	0.75 (0.12)	1.175	0.184	
Ambiguous SRGP rate (mean, S.D.)	0.20 (0.20)	0.21 (0.19)	0.037 (0.042)	37.623	** < 0.001**	FES > HC***, ARMS > HC***
Unambiguous SRGP rate (mean, S.D.)	0.14 (0.19)	0.10 (0.16)	0.01 (0.02)	23.639	** < 0.001**	FES > HC***, ARMS > HC***

*PANSS* Positive and Negative Syndrome Scale for psychotic symptoms, *DDD* daily dosage, *DUI* duration of illness, *IRIS* idea of reference interview scale, *LSAS* Liebowitz social anxiety scale, *PDI* Peter’s delusion inventory, *S.D.* standard deviations, *SRGP* self-referential gaze perception, *F* One-way ANOVA, χ^*2*^ Kruskal–Wallis H Test, *U* Mann–Whitney U test

Significant level at *^p* < 0.10

*p < 0.05

**p < 0.01

***p < 0.00

### Comparison of cognitive and social cognitive performance

One-way ANOVA suggested significant three group differences in executive functions (*F* = 26.401, *p* < 0.001), processing speed (*F* = 25.212, *p* < 0.001), working memory (*F* = 22.697, *p* < 0.001), and overall neurocognition (*F* = 51.553, *p* < 0.001). Post-hoc pairwise comparisons across neurocognitive domains indicated that HC performed better than FES and ARMS individuals (*p’s* < 0.001) with no significant differences between ARMS and FES (*p’s* = 0.144–0.995) (Table 1).

**Fig. 1:**
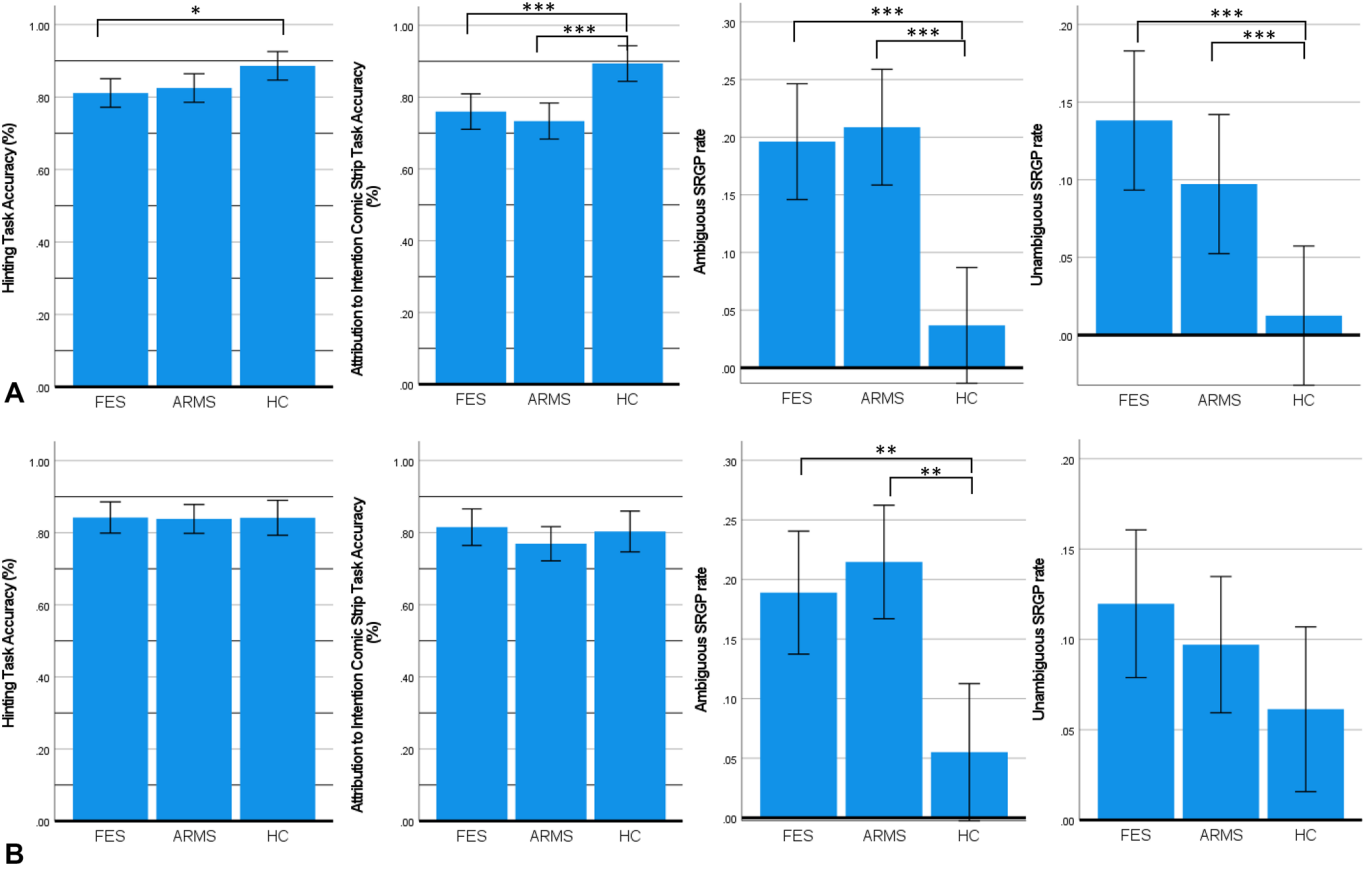
**A** Comparisons of performances in the hinting task, comic strip task, and gaze perception task between the first-episode schizophrenia spectrum disorder (FES), at-risk mental states (ARMS), and healthy control (HC) conditions using ANOVA. **B** Controlled comparisons of mentalizing measures between conditions with age, gender, years of education, and neurocognition as covariates using ANCOVA. Post hoc pairwise comparisons were used with Bonferroni corrections to examine significant group differences. Significant level at *p < 0.05, **p < 0.01, ***p < 0.001. Error bars represent the standard error of the mean

Significant three group differences were observed in the comic strip task (*F* = 11.991, *p* < 0.001), hinting task (*F* = 4.028, *p* = 0.020), ambiguous SRGP rate (Wald *χ*^*2*^ = 37.623, *p* < 0.001), and unambiguous SRGP rate (Wald χ^*2*^ = 23.639, *p* < 0.001). Pairwise comparisons further suggested significant differences between HC with FES (*p* < 0.001) and ARMS (*p* < 0.001). However, only FES patients performed significantly worse than HC in the hinting task (*p* = 0.026), while there was no significant difference between ARMS and HC (*p* = 0.094). No significant differences were found between FES and ARMS participants across all social cognitive tasks (*p’s* = 0.609–1.000) (Table 1). However, after controlling for demographics and neurocognition, only ambiguous SRGP rate remained significantly different between three groups (*F* = 6.151, *p* < 0.001, *Partial η*^*2*^ = 0.098), while no significant three group differences in the comic strip and hinting tasks was found (Table 2). Pairwise comparisons revealed significant differences between HC with FES (*p* = 0.009) and ARMS (*p* = 0.002) (Fig. 1).

**Table 2:** ANCOVA for social cognition across FES, ARMS, and control groups

Mentalizing measures^a^	*F*	*p* value	*Partial η*^*2*^	Pairwise comparison	
				Group comparisons	*p value*
Comic strip task	0.618	0.541	0.011		
Hinting task	0.297	0.744	0.005		
Ambiguous SRGP rate	6.151	0.003	0.098	FES > HC	**0.009**
Unambiguous SRGP rate	0.803	0.451	0.014	ARMS > HC	**0.002**
				ARMS > FES	1.000

SRGP self-referential gaze perception

^a^Univariate analyses controlling age, gender, years of education, and cognitive composite scores were conducted. Values of mean and standard error are adjusted with covariates. The bold values are significant p values

### Correlations and regression analysis

Table 3 displayed the correlations between performances of the three tasks with clinical and neurocognitive variables for each group. No significant relationship between cognitive functions with DDD, DUI, and DUP was found in ARMS and FES. Only significant variables were included in the multiple regression analysis. For FES patients, executive functions had a significant relationship with the comic strip task, accounting for 36.8% of the variance (Table 4). Executive functions and PANSS positive symptoms significantly explained 20.1% and 27.2% of the variance in ambiguous and unambiguous SRGP rates respectively.

**Table 3:** Correlations between social cognition and basic demographics, clinical characteristics, and neurocognition

Groups	*FES*				*ARMS*				*HC*			
Variables	Comic strip task	Hinting Task	Ambiguous SRGP rate	Unambiguous SRGP rate	Comic strip task	Hinting Task	Ambiguous SRGP rate	Unambiguous SRGP rate	Comic strip task	Hinting Task	Ambiguous SRGP rate	Unambiguous SRGP rate
Demographic												
Age	0.150	0.112	− 0.114	− 0.136	0.115	0.144	0.157	− 0.104	0.261	− 0.047	− 0.185	− 0.264^
Gender	0.210	0.155	0.009	0.103	− 0.130	− 0.119	0.028	0.250	0.138	0.034	0.105	− 0.004
Years of education	0.107	0.110	− 0.286	− 0.332*	0.390^*^	0.157	− 0.162	− 0.284^	0.349^*^	0.082	− 0.295^	− 0.354*
DDD	0.011	0.152	0.009	0.022	0.113	0.155	0.052	0.170	N/A	N/A	N/A	N/A
DUI	– 0.015	0.077	0.108	0.108	N/A	N/A	N/A	N/A	N/A	N/A	N/A	N/A
DUP	– 0.048	0.211	0.248	0.249	N/A	N/A	N/A	N/A	N/A	N/A	N/A	N/A
Clinical characteristics												
PANSS positive	0.051	0.107	0.314*	0.348^*^	0.100	0.283^	– 0.069	0.239	N/A	N/A	N/A	N/A
PANSS negative	– 0.002	– 0.229	0.051	0.134	0.006	– 0.231	– 0.019	– 0.033	N/A	N/A	N/A	N/A
PANSS disorganization	– 0.344*	– 0.161	0.130	0.223	0.277^	– 0.017	– 0.247	– 0.077	N/A	N/A	N/A	N/A
PANSS depression– anxiety	0.116	– 0.061	0.001	0.150	0.306^	0.122	0.208	0.119	N/A	N/A	N/A	N/A
PANSS excitement/activity	– 0.190	– 0.188	0.024	0.170	0.107	0.001	– 0.218	– 0.246	N/A	N/A	N/A	N/A
IRIS item	0.159	– 0.083	0.101	0.058	– 0.020	0.201	– 0.094	0.272^	0.060	– 0.198	0.408^**^	0.393^*^
LSAS total	0.147	– 0.087	– 0.117	– 0.079	– 0.233	– 0.059	0.582^***^	0.681^***^	– 0.156	– 0.168	0.146	0.313^*^
PDI total	– 0.153	0.013	0.293^	0.318^	– 0.126	– 0.097	0.368^*^	0.431^**^	– 0.112	– 0.195	– 0.092	– 0.062
Neurocognition												
Executive functions	0.579**	0.140	– 0.420**	– 0.453**	0.308^	0.185	– 0.060	– 0.226	– 0.197	0.126	0.193	– 0.270^
Processing speed	0.265^	0.145	– 0.135	– 0.191	0.392*	0.224	– 0.427**	– 0.494**	0.203	0.213	0.000	0.089
Working memory	0.045	– 0.079	– 0.088	– 0.210	0.498**	0.297^	– 0.109	– 0.336*	0.191	– 0.025	0.108	0.053
Cognitive composite score	0.452**	0.124	– 0.314*	– 0.404**	0.486**	0.285^	– 0.286^	– 0.456**	0.099	0.142	0.141	– 0.054

*PANSS* Positive and Negative Syndrome Scale for psychotic symptoms, *IRIS* idea of reference interview scale, *LSAS* Liebowitz social anxiety scale, *PDI* Peter’s delusion inventory, *DDD* defined daily dose, *DUI* duration of illness, *SRGP* self-referential gaze perception

Significant level at ^*p* < 0.1

*p < 0.05

**p < 0.01

***p < 0.001

**Table 4:** Multiple regression analysis of cognition controlling age, gender, years of education

Groups	Mentalizing measures	Predictor	*β*	*t*	*p*	Model properties
*FES*	Comic strip task	**Executive functions**	0.574	3.868	< .001***	*F* = 5.541, *p* < **0.001**, R^2^ = 0.449, adj. R^2^ = 0.368
		PANSS disorganization	– 0.127	– 0.927	0.361	
	Hinting task	N/A				
	Ambiguous SRGP rate	**Executive functions**	– 0.349	– 2.25	0.031*	*F* = 2.964, *p* = **0.025**, R^2^ = 0.304, adj. R^2^ = 0.201
		**PANSS positive**	0.305	2.066	0.047*	
	Unambiguous SRGP rate	**Executive functions**	– 0.362	– 2.446	0.020*	*F* = 3.917, *p* = **0.007**, R^2^ = 0.365, adj. R^2^ = 0.272
		**PANSS positive**	0.345	2.449	0.038*	
*ARMS*	Comic strip task	Processing speed	0.195	1.212	0.234	*F* = 4.197, *p* = **0.004**, R^2^ = 0.382, adj. R^2^ = 0.291
		**Working memory**	0.363	2.101	0.043*	
	Hinting task	N/A				
	Ambiguous SRGP rate	Processing speed	– 0.165	– 1.082	0.287	*F* = 4.296, *p* = **0.003**, R^2^ = 0.439, adj. R^2^ = 0.336
		**LSAS total**	0.459	2.625	0.013*	
		PDI total	0.071	0.422	0.675	
	Unambiguous SRGP rate	Processing speed	– 0.169	– 1.178	0.248	*F* = 7.793, *p* < **0.001**, R^2^ = 0.630, adj. R^2^ = 0.549
		Working memory	– 0.115	– 0.835	0.410	
		**LSAS total**	0.598	4.152	< .001***	
		PDI total	– 0.019	– 0.135	0.893	
*HC*	Comic strip task	N/A				
	Hinting task	N/A				
	Ambiguous SRGP rate	**IRIS item**	0.374	2.441	0.020*	*F* = 2.571, *p* = 0.055, R^2^ = 0.227, adj. R^2^ = 0.139
	Unambiguous SRGP rate	IRIS ietm	0.317	1.898	0.066^	*F* = 2.313, *p* = 0.065, R^2^ = 0.254, adj. R^2^ = 0.144
		LSAS total	0.060	0.337	0.738	

*PANSS* Positive and Negative Syndrome Scale for psychotic symptoms, *IRIS* idea of reference interview scale, *LSAS* Liebowitz social anxiety scale, *PDI* Peter’s delusion inventory, *SRGP* self-referential gaze perception

Significant level at

*^p* < 0.10

^*^p < 0.05

^**^p < 0.01

^***^p < 0.001

For ARMS participants, working memory significantly accounted for 29.1% of the variance in the comic strip task. Regarding SRGP rates, 33.6% of the variance in ambiguous SRGP rate could be explained by processing speed, LSAS, and PDI, while processing speed, working memory, LSAS, and PDI could account for 53.5% of the variance in unambiguous SRGP rate (Table 4). LSAS total score was the most significant variable in the regression models for both ambiguous SRGP rate (*β* = 0.459, *p* = 0.013) and unambiguous SRGP rate (*β* = 0.598, *p* < 0.001).

Additionally, correlations in the full sample indicated that the four social cognitive measures were significantly associated with the three neurocognitive domains (Supplementary Table 1). The social cognitive measures, except the hinting task, were also significantly related to years of education, LSAS, and PDI. Ambiguous and unambiguous SRGP rates were positively correlated with IRIS. For FES and ARMS participants, PANSS positive and negative symptoms were related to the unambiguous SRGP rate and hinting task respectively.

## Discussion

The current study incorporated three social cognitive tasks to compare the verbal and non-verbal mentalizing abilities, and hypermentalizing tendency between patients with FES, individuals with ARMS, and HC. Ambiguous and unambiguous SRGP rates in the gaze perception task, and the comic strip task were significantly impaired in both FES and ARMS compared to HC, whereas the hinting task was only significantly worse in FES compared to HC. ARMS and FES did not significantly differ among the three tasks. However, after controlling for demographics and general neurocognition, only ambiguous SRGP rate remained significantly higher in FES and ARMS participants than that of the HC. Although ARMS and FES participants displayed similar behavioral deficits, distinct stage-specific relationships between social cognitive impairments with symptomatology and neurocognitive domains were identified in the two groups. Specifically, impairments were significantly associated with social anxiety, processing speed, and working memory in ARMS, while executive functions and positive symptoms played significant roles in FES. To our best knowledge, the present study is the first to investigate self-referential hypermentalizing bias in ARMS individuals and its relationships with neurocognition and symptoms.

The verbal mentalizing impairments as measured by the hinting task in FES compared to HC may be due to the greater severity of positive and disorganization symptoms of FES hampering social cognition [13], whereas ARMS only exhibited a trend toward significance compared to HC. The comparable difficulties in non-verbal mentalizing in ARMS and FES participants found in the current study is consistent with previous literature [10]. It may be attributable to the fact that the ARMS participants in this study were a help-seeking population who exhibited similar levels of neurocognitive impairments and subclinical features, which was also evident by the relatively large proportion of ARMS individuals prescribed with antipsychotics (52.5%) [54]. Furthermore, after accounting for the effects of demographics and cognitive functions, the three groups no longer differed in the both verbal and non-verbal mentalizing abilities (Fig. 1). This was compatible with previous studies that social cognitive or mentalizing impairments in FES and ARMS individuals could be primarily explained by neurocognitive impairments [24]. However, the ambiguous SRGP rate in FES and ARMS remained significantly higher than HC even after controlling for the same covariates with a moderate to large effect size (*Partial η*^*2*^ = 0.098). These results suggested that the self-referential biases of gazes may be a distinct characteristic at the early-stage and prodromal psychosis. Misinterpreting ambiguous gazes of others as self-directing gazes could be prominent manifestations in FES and ARMS individuals, reflecting some core features of psychotic illness which were not likely to be explained by the neurocognitive impairments. Hence, in addition to the conventional self-report questionnaires and clinical interviews [55], self-referential hypermentalizing bias may serve as a complementary behavioral indicator to identify subclinical individuals with attenuated psychotic symptoms.

Despite the lack of differences in neurocognitive impairments, mentalizing abilities, and self-referential hypermentalizating errors between FES and ARMS, differential associations between the social cognitive impairments with neurocognitive domains and symptoms were identified. These findings offered insights into potential stage-specific mechanisms or pathways towards the social cognitive impairments for individuals at the different stages of psychosis, thus extending our understanding of the complex nature of the impairments. For neurocognitive domains, correlation and regression analyses indicated that higher-order executive functions were significantly associated with non-verbal mentalizing ability and self-referential hypermentalizing bias in FES patients. In contrast, processing speed and working memory played significant roles in non-verbal mentalizing ability and hypermentalizing biases, while there was also a trend significant association between verbal mentalizing ability and working memory in ARMS individuals (Table 2). These associations between social cognitive impairments and different neurocognitive domains in FES and ARMS were consistent with previous findings [16–19]. Neuroimaging studies have also provided concordant evidence of differences in neural activation profiles during mentalizing tasks between individuals with schizophrenia and ARMS [56, 57], including regions in the superior temporal sulcus (STS), inferior frontal gyrus (IFG), and temporoparietal junction (TPJ). Altogether, the results highlight the distinct contributions of neurocognitive domains to the social cognitive functions in FES and ARMS, and thus comprehensive assessments and personalized interventions in neurocognition and social cognitions for individuals at different stages of psychotic illness could be developed to optimize treatment efficacy and result in better long-term outcomes.

When examining the FES and ARMS as a group, negative symptoms were only related to the verbal mentalizing ability suggesting the presence of different mechanisms in social cognition domains. The lack of significant relationship between negative symptoms and social cognition when examining the clinical groups separately may be attributed by a lack of power due to smaller sample size. Positive symptoms were related to unambiguous gaze perception bias when examining the FES and ARMS as a group. Furthermore, positive symptoms were particularly associated with the hypermentalizing bias with ambiguous and unambiguous gazes in the FES population, consistent with previous works [25, 26, 51]. Misjudging intentions from others was featured in the initial stage of delusion formation, where individuals could not effectively handle social ambiguity and jump into conclusions with biased perceptions and interpretations [58]. On the other hand, findings herein suggested the associations between self-referential judgements with subclinical delusional ideations and social anxiety in ARMS, where the latter played a prominent role in both ambiguous (*β* = 0.459) and unambiguous (*β* = 0.598) gazes. Align with the prior study on CHR [33], social anxiety has been linked to social cognitive biases and impairments, with affected individuals often feeling discomfort and fear during social interactions and gaze perception, leading to irregular gaze patterns and avoidance behaviors [29, 30, 59, 60]. Additionally, a neuroimaging study by Ahrens et al. [61] showed that anxious individuals had impaired cortical activation in distinguishing irrelevant social stimuli. Along with the substantial prevalence of anxiety symptoms in ARMS individuals [31], these findings not only underlined the critical need to focus on social anxiety in therapeutic interventions but also highlighted the heterogeneity of psychopathologies in the ARMS population. Indeed, social anxiety, referential ideation, and paranoid ideation were found to be related with social cognition across the patient, subclinical, and healthy control participants. These results suggested the importance of trait-like features in social cognition and functioning deficits and the need to consider a broad spectrum of transdiagnostic and subclinical symptoms to tailor more effective and personalized treatment strategies in the prodromal stage, improving social functioning and mitigating the risk of progression to more severe psychiatric conditions.

One of the limitations of the current study was the small sample size which might have limited the statistical power of the study to detect some possible associations. Second, our ARMS sample only included help-seeking individuals from the psychiatric services, and a relatively large proportion were medicated with antipsychotics. Therefore, the results might not be generalizable to community or non-help-seeking samples. However, the DDD of antipsychotics was not found to be related to any social cognitive deficit in ARMS (Table 3). Third, our study excluded individuals with mood and anxiety disorders to minimize confounding factors and focus on the link between mentalizing and schizophrenia-specific symptoms. This may limit the generalizability of our results. Future studies should consider including these populations to have better representation of clinical diversity and investigate the relationship between affective symptoms and mentalizing. Forth, other variables that have been suggested to be associated with social cognitive functions were not included in this study, such as autistic traits [62] and social motivation [63]. Additionally, this was a cross-sectional study and predictive links could not be inferred. Therefore, a longitudinal study with ARMS individuals could explore the trajectories of social cognition across the early stages of psychosis development and the role of mentalizing abilities in predicting outcomes. Additionally, we acknowledge that the conceptualization of mentalizing or ToM was still an ongoing debate and the mentalizing tasks in general often suffered from insufficient psychometric evaluations and doubtful validity [11, 64, 65]. As such we chose to utilize the two mentalizing tasks with distinct operationalizations in our study where both had demonstrated relatively good face validity and psychometric performance in our samples and in other large-scale studies [66]. Future studies should also examine other dimensions of mentalizing and self-referential bias, such as affective and implicit mentalizing and perception of self-referential gestures, for a more comprehensive understanding of social cognitive impairments in the early-stage and prodromal psychosis.

The current study indicated that FES patients and ARMS individuals displayed comparable patterns of mentalizing impairments and self-referential hypermentalizing bias in controlled comparisons. Only ambiguous SRGP rate (self-referential hypermentalizing errors) was found to be significantly higher in both FES and ARMS compared to HC after controlling for the basic demographics and neurocognition, and thus may be considered as a behavioral sign in the early and prodromal psychosis. Executive functions and positive symptoms were significantly related to social cognitive impairments in the FES group, while working memory and social anxiety were found to play a prominent role in mentalizing deficits and self-referential bias in ARMS individuals. These results identify stage-specific relationships between social cognitive impairments with neurocognitive domains and social anxiety, suggesting possible therapeutic targets for personalized interventions to improve social cognition and psychosocial functioning.

## Supplementary Information

Below is the link to the electronic supplementary material.

Supplementary file1 (DOCX 110 KB)

Supplementary file2 (DOCX 22 KB)

## Data Availability

The data that support the findings of this study are available on request from the corresponding author. The data are not publicly available due to potential privacy issues.

## References

[1] Frith CD, Frith U (2012) Mechanisms of social cognition. Annu Rev Psychol 63:287–313. 10.1146/annurev-psych-120710-100449 21838544 10.1146/annurev-psych-120710-100449

[2] Premack D, Woodruff G (1978) Does the chimpanzee have a theory of mind? Behavioral and Brain Sciences 1:515

[3] Sprong M, Schothorst P, Vos E et al (2007) Theory of mind in schizophrenia. Br J Psychiatry 191:5–13. 10.1192/bjp.bp.107.035899 17602119 10.1192/bjp.bp.107.035899

[4] Healey KM, Bartholomeusz CF, Penn DL (2016) Deficits in social cognition in first episode psychosis: a review of the literature. Clin Psychol Rev 50:108–137. 10.1016/j.cpr.2016.10.001 27771557 10.1016/j.cpr.2016.10.001

[5] Couture SM, Penn DL, Roberts DL (2006) The functional significance of social cognition in schizophrenia: a review. Schizophr Bull 32:S44–S63. 10.1093/schbul/sbl029 16916889 10.1093/schbul/sbl029 PMC2632537

[6] Bora E, Pantelis C (2013) Theory of mind impairments in first-episode psychosis, individuals at ultra-high risk for psychosis and in first-degree relatives of schizophrenia: systematic review and meta-analysis. Schizophr Res 144:31–36. 10.1016/j.schres.2012.12.013 23347949 10.1016/j.schres.2012.12.013

[7] Lee TY, Hong SB, Shin NY, Kwon JS (2015) Social cognitive functioning in prodromal psychosis: a meta-analysis. Schizophr Res 164:28–34. 10.1016/j.schres.2015.02.008 25749019 10.1016/j.schres.2015.02.008

[8] van Donkersgoed RJM, Wunderink L, Nieboer R et al (2015) Social cognition in individuals at ultra-high risk for psychosis: a meta-analysis. PLoS ONE 10:e0141075. 10.1371/journal.pone.0141075 26510175 10.1371/journal.pone.0141075 PMC4624797

[9] Kuis DJ, van de Giessen T, de Jong S et al (2021) Empathy and its relationship with social functioning in individuals at ultra-high risk for psychosis. Front Psychiatry. 10.3389/fpsyt.2021.730092 34858222 10.3389/fpsyt.2021.730092 PMC8632546

[10] Ntouros E, Karanikas E, Floros G et al (2018) Social cognition in the course of psychosis and its correlation with biomarkers in a male cohort. Cogn Neuropsychiatry 23:103–115. 10.1080/13546805.2018.1440201 29447074 10.1080/13546805.2018.1440201

[11] Yeh YC, Lin CY, Li PC et al (2021) A systematic review of the current measures of theory of mind in adults with schizophrenia. Int J Environ Res Public Health 18:7172–7172. 10.3390/ijerph18137172 34281109 10.3390/ijerph18137172 PMC8297277

[12] Thibaudeau É, Achim AM, Parent C et al (2020) A meta-analysis of the associations between theory of mind and neurocognition in schizophrenia. Schizophr Res 216:118–128. 10.1016/j.schres.2019.12.017 31899095 10.1016/j.schres.2019.12.017

[13] Thibaudeau E, Rae J, Raucher-Chéné D et al (2023) Disentangling the relationships between the clinical symptoms of schizophrenia spectrum disorders and theory of mind: a meta-analysis. Schizophr Bull 49:255–274. 10.1093/schbul/sbac150 36244001 10.1093/schbul/sbac150 PMC10016420

[14] Green MF, Bearden CE, Cannon TD et al (2012) Social cognition in schizophrenia, part 1: performance across phase of illness. Schizophr Bull 38:854–864. 10.1093/schbul/sbq171 21345917 10.1093/schbul/sbq171 PMC3406534

[15] Ayesa-Arriola R, Setién-Suero E, Neergaard KD et al (2016) Evidence for trait related theory of mind impairment in first episode psychosis patients and its relationship with processing speed: a 3 year follow-up study. Front Psychol 7:592. 10.3389/fpsyg.2016.00592 27199826 10.3389/fpsyg.2016.00592 PMC4854883

[16] Catalan A, Angosto V, Díaz A et al (2018) The relationship between theory of mind deficits and neurocognition in first episode-psychosis. Psychiatry Res 268:361–367. 10.1016/j.psychres.2018.06.066 30099276 10.1016/j.psychres.2018.06.066

[17] Fernandez-Gonzalo S, Jodar M, Pousa E et al (2014) Selective effect of neurocognition on different theory of mind domains in first-episode psychosis. J Nervous Mental Dis 202:576–582. 10.1097/nmd.0000000000000164 10.1097/NMD.0000000000000164 25010103

[18] Zhang T, Yi Z, Li H et al (2016) Faux pas recognition performance in a help-seeking population at clinical high risk of psychosis. Eur Arch Psychiatry Clin Neurosci 266:71–78. 10.1007/s00406-015-0615-z 26189033 10.1007/s00406-015-0615-z

[19] Zhang T, Xu L-H, Cui H et al (2018) Changes in correlation characteristics of time consumption and mind-reading performance in pre-onset and post-onset psychosis. Psychiatry Res 262:168–174. 10.1016/j.psychres.2018.02.008 29453035 10.1016/j.psychres.2018.02.008

[20] Chung YS, Kang DH, Shin NY et al (2008) Deficit of theory of mind in individuals at ultra-high-risk for schizophrenia. Schizophr Res 99:111–118. 10.1016/j.schres.2007.11.012 18096371 10.1016/j.schres.2007.11.012

[21] Kong W, Koo SJ, Seo E et al (2021) Empathy and theory of mind in ultra-high risk for psychosis: relations with schizotypy and executive function. Psychiatry Investig. 18:1109–1116. 10.30773/pi.2021.0111 34710958 10.30773/pi.2021.0111 PMC8600219

[22] Yong E, Barbato M, Penn DL et al (2014) Exploratory analysis of social cognition and neurocognition in individuals at clinical high risk for psychosis. Psychiatry Res 218:39–43. 10.1016/j.psychres.2014.04.003 24755041 10.1016/j.psychres.2014.04.003 PMC4062969

[23] Ohmuro N, Katsura M, Obara C et al (2016) Deficits of cognitive theory of mind and its relationship with functioning in individuals with an at-risk mental state and first-episode psychosis. Psychiatry Res 243:318–325. 10.1016/j.psychres.2016.06.051 27434201 10.1016/j.psychres.2016.06.051

[24] Hur J-W, Byun MS, Shin NY et al (2013) General intellectual functioning as a buffer against theory-of-mind deficits in individuals at ultra-high risk for psychosis. Schizophr Res 149:83–87. 10.1016/j.schres.2013.06.019 23810120 10.1016/j.schres.2013.06.019

[25] Chan SKW, Liu T, Wong AOY et al (2021) Self-referential gaze perception of patients with schizophrenia and its relationship with symptomatology and cognitive functions. Schizophr Res 228:288–294. 10.1016/j.schres.2020.12.034 33493777 10.1016/j.schres.2020.12.034

[26] Fretland RA, Andersson S, Sundet K et al (2015) Theory of mind in schizophrenia: error types and associations with symptoms. Schizophr Res 162:42–46. 10.1016/j.schres.2015.01.024 25623602 10.1016/j.schres.2015.01.024

[27] Peyroux E, Prost Z, Danset-Alexandre C et al (2019) From “under” to “over” social cognition in schizophrenia: Is there distinct profiles of impairments according to negative and positive symptoms? Schizophr Res Cogn 15:21–29. 10.1016/j.scog.2018.10.001 30534527 10.1016/j.scog.2018.10.001 PMC6260279

[28] White TP, Borgan F, Ralley O, Shergill SS (2016) You looking at me?: Interpreting social cues in schizophrenia. Psychol Med 46:149–160. 10.1017/s0033291715001622 26338032 10.1017/S0033291715001622

[29] Ballespí S, Vives J, Sharp C et al (2019) Hypermentalizing in social anxiety: evidence for a context-dependent relationship. Front Psychol 10:1501. 10.3389/fpsyg.2019.01501 31354562 10.3389/fpsyg.2019.01501 PMC6629962

[30] Chen J, Short M, Kemps E (2020) Interpretation bias in social anxiety: a systematic review and meta-analysis. J Affect Disord 276:1119–1130. 10.1016/j.jad.2020.07.121 32777650 10.1016/j.jad.2020.07.121

[31] Deng W, Addington J, Bearden CE et al (2022) Characterizing sustained social anxiety in individuals at clinical high risk for psychosis: trajectory, risk factors, and functional outcomes. Psychol Med. 10.1017/s0033291722000277 35144716 10.1017/S0033291722000277 PMC10277760

[32] Michail M, Birchwood M (2009) Social anxiety disorder in first-episode psychosis: incidence, phenomenology and relationship with paranoia. Br J Psychiatry 195:234–241. 10.1192/bjp.bp.108.053124 19721113 10.1192/bjp.bp.108.053124

[33] Williams TF, Conley RE, Mittal VA (2023) The relevance of social anxiety for understanding social functioning and facial emotion recognition in individuals at clinical high-risk for psychosis. Early Interv Psychiatry 17:1021–1027. 10.1111/eip.13396 36641807 10.1111/eip.13396 PMC10349169

[34] Chan SKW, So HC, Hui CLM et al (2015) 10-year outcome study of an early intervention program for psychosis compared with standard care service. Psychol Med 45:1181–1193. 10.1017/s0033291714002220 25233868 10.1017/S0033291714002220

[35] American Psychiatric Association (2013) Diagnostic and statistical manual of mental disorders: DSM-5 (5th edition). Ref Rev. 10.1108/rr-10-2013-0256

[36] Yung AR, Yuen HP, McGorry PD et al (2005) Mapping the onset of psychosis: the comprehensive assessment of at-risk mental states. Aust N Z J Psychiatry 39:964–971. 10.1080/j.1440-1614.2005.01714.x 16343296 10.1080/j.1440-1614.2005.01714.x

[37] Peters ER, Joseph SA, Garety PA (1995) The measurement of delusional ideation in the normal population—introducing the PDI (PEters et al. delusions inventory). Schizophr Res. 15:19. 10.1016/0920-9964(95)95071-g 10.1093/oxfordjournals.schbul.a033401 10478789

[38] Wong GHY, Hui CLM, Tang JYM et al (2012) Screening and assessing ideas and delusions of reference using a semi-structured interview scale: a validation study of the Ideas of Reference Interview Scale (IRIS) in early psychosis patients. Schizophr Res 135:158–163. 10.1016/j.schres.2011.12.006 22244183 10.1016/j.schres.2011.12.006

[39] Heimberg RG, Horner KJ, Juster HR et al (1999) Psychometric properties of the Liebowitz Social Anxiety Scale. Psychol Med 29:199–212. 10.1017/s0033291798007879 10077308 10.1017/s0033291798007879

[40] Kay SR, Fiszbein A, Opler LA (1987) The Positive and Negative Syndrome Scale (PANSS) for Schizophrenia. Schizophr Bull 13:261–276. 10.1093/schbul/13.2.261 3616518 10.1093/schbul/13.2.261

[41] Häfner H, Riecher-Rössler A, Hambrecht M et al (1992) IRAOS: an instrument for the assessment of onset and early course of schizophrenia. Schizophr Res 6:209–223. 10.1016/0920-9964(92)90004-o 1571314 10.1016/0920-9964(92)90004-o

[42] Leucht S, Samara M, Heres S, Davis JM (2016) Dose equivalents for antipsychotic drugs: the DDD method: table 1. Schizophr Bull 42:S90–S94. 10.1093/schbul/sbv167 27460622 10.1093/schbul/sbv167 PMC4960429

[43] Silverstein AB (1982) Two- and four-subtest short forms of the Wechsler Adult Intelligence Scale-Revised. J Consult Clin Psychol 50:415–418. 10.1037/0022-006x.50.3.415

[44] Sánchez-Cubillo I, Periáñez JA, Adrover-Roig D et al (2009) Construct validity of the Trail Making Test: role of task-switching, working memory, inhibition/interference control, and visuomotor abilities. J Int Neuropsychol Soc 15:438–450. 10.1017/s1355617709090626 19402930 10.1017/S1355617709090626

[45] Brunet E, Sarfati Y, Hardy-Baylé M-C (2003) Reasoning about physical causality and other’s intentions in schizophrenia. Cogn Neuropsychiatry 8:129–139. 10.1080/13546800244000256 16571555 10.1080/13546800244000256

[46] Sarfati Y, Hardy-Baylé M, Besche C, Widlöcher D (1997) Attribution of intentions to others in people with schizophrenia: a non-verbal exploration with comic strips. Schizophr Res 25:199–209. 10.1016/s0920-9964(97)00025-x 9264175 10.1016/s0920-9964(97)00025-x

[47] Corcoran R, Mercer G, Frith CD (1995) Schizophrenia, symptomatology and social inference: investigating “theory of mind” in people with schizophrenia. Schizophr Res 17:5–13. 10.1016/0920-9964(95)00024-g 8541250 10.1016/0920-9964(95)00024-g

[48] Klein HS, Springfield CR, Bass E et al (2020) Measuring mentalizing: a comparison of scoring methods for the hinting task. Int J Methods Psychiatric Res. 10.1002/mpr.1827 10.1002/mpr.1827 PMC7301277 32385868

[49] Terwee CB, Bot SDM, de Boer MR et al (2007) Quality criteria were proposed for measurement properties of health status questionnaires. J Clin Epidemiol 60:34–42. 10.1016/j.jclinepi.2006.03.012 17161752 10.1016/j.jclinepi.2006.03.012

[50] George D, Mallery P (2019) IBM SPSS statistics 26 step by step: a simple guide and reference. Routledge, New York, London

[51] Chan SKW, Hsiao J, Wong AOY et al (2022) Explicit and implicit mentalization of patients with first-episode schizophrenia: a study of self-referential gaze perception with eye movement analysis using hidden Markov models. Eur Arch Psychiatry Clin Neurosci 272:1335–1345. 10.1007/s00406-022-01383-y 35079856 10.1007/s00406-022-01383-y

[52] Shafer A, Dazzi F (2019) Meta-analysis of the positive and Negative Syndrome Scale (PANSS) factor structure. J Psychiatr Res 115:113–120. 10.1016/j.jpsychires.2019.05.008 31128501 10.1016/j.jpsychires.2019.05.008

[53] Sakia RM (1992) The Box-Cox transformation technique: a review. The Statistician 41:169. 10.2307/2348250

[54] Schultze-Lutter F, Rahman J, Ruhrmann S et al (2015) Duration of unspecific prodromal and clinical high risk states, and early help-seeking in first-admission psychosis patients. Soc Psychiatry Psychiatr Epidemiol 50:1831–1841. 10.1007/s00127-015-1093-3 26155901 10.1007/s00127-015-1093-3

[55] Addington J, Stowkowy J, Weiser M (2015) Screening tools for clinical high risk for psychosis. Early Interv Psychiatry 9:345–356. 10.1111/eip.12193 25345316 10.1111/eip.12193

[56] Ilzarbe D, Baeza I, de la Serna E et al (2021) Theory of mind performance and prefrontal connectivity in adolescents at clinical high risk for psychosis. Dev Cogn Neurosci 48:100940. 10.1016/j.dcn.2021.100940 33721828 10.1016/j.dcn.2021.100940 PMC7970321

[57] Vucurovic K, Caillies S, Kaladjian A (2021) Neural correlates of mentalizing in individuals with clinical high risk for schizophrenia: ALE meta-analysis. Front Psychiatry. 10.3389/fpsyt.2021.634015 33959048 10.3389/fpsyt.2021.634015 PMC8095711

[58] Diaconescu AO, Hauke DJ, Borgwardt S (2019) Models of persecutory delusions: a mechanistic insight into the early stages of psychosis. Mol Psychiatry 24:1258–1267. 10.1038/s41380-019-0427-z 31076646 10.1038/s41380-019-0427-z PMC6756090

[59] Schulze L, Renneberg B, Lobmaier JS (2013) Gaze perception in social anxiety and social anxiety disorder. Front Hum Neurosci 7:872. 10.3389/fnhum.2013.00872 24379776 10.3389/fnhum.2013.00872 PMC3863960

[60] Wastler HM, Lenzenweger MF (2019) Self-referential hypermentalization in schizotypy. Personal Disord Theory Res Treat 10:536–544. 10.1037/per0000344 10.1037/per0000344 31144838

[61] Ahrens LM, Mühlberger A, Pauli P, Wieser MJ (2014) Impaired visuocortical discrimination learning of socially conditioned stimuli in social anxiety. Soc Cogn Affect Neurosci 10:929–937. 10.1093/scan/nsu140 25338634 10.1093/scan/nsu140 PMC4483562

[62] Foss-Feig JH, Velthorst E, Smith L et al (2019) Clinical profiles and conversion rates among young individuals with autism spectrum disorder who present to clinical high risk for psychosis services. J Am Acad Child Adolesc Psychiatry 58:582–588. 10.1016/j.jaac.2018.09.446 30797038 10.1016/j.jaac.2018.09.446 PMC7781438

[63] Lecce S, Ceccato I, Bianco F et al (2015) Theory of Mind and social relationships in older adults: the role of social motivation. Aging Ment Health 21:253–258. 10.1080/13607863.2015.1114586 26581839 10.1080/13607863.2015.1114586

[64] Konstantin GE, Nordgaard J, Henriksen MG (2023) Methodological issues in social cognition research in autism spectrum disorder and schizophrenia spectrum disorder: a systematic review. Psychol Med 53:3281–3292. 10.1017/s0033291723001095 37161884 10.1017/S0033291723001095 PMC10277762

[65] Quesque F, Rossetti Y (2020) What do theory-of-mind tasks actually measure? Theory and practice. Perspect Psychol Sci 15:384–396. 10.1177/1745691619896607 32069168 10.1177/1745691619896607

