# Supplementary figures and images for: Mentalizing impairments and hypermentalizing bias in individuals with first-episode schizophrenia-spectrum disorder and at-risk mental state: the differential roles of neurocognition and social anxiety
Source: Eur Arch Psychiatry Clin Neurosci. 2024 Jul 3;275(3):907–19. doi: 10.1007/s00406-024-01830-y (PMC11947045; doi:10.1007/s00406-024-01830-y)

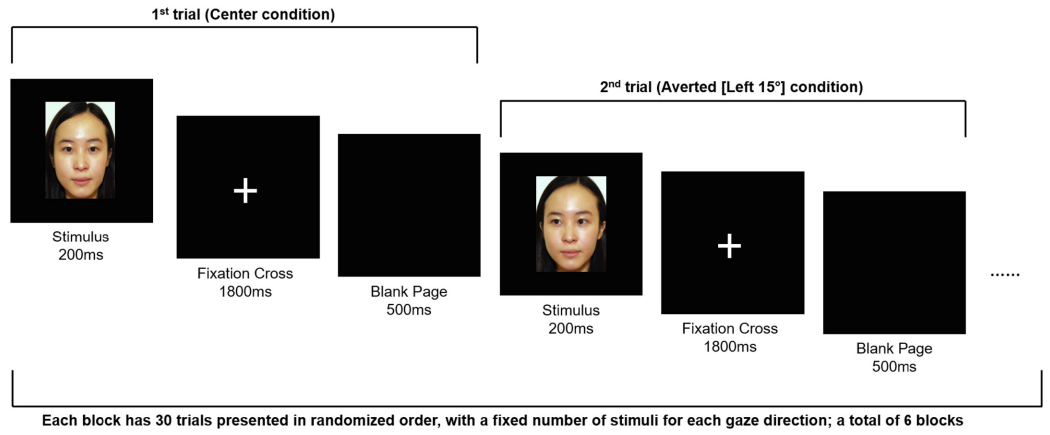


Supplementary Figure 1. Experimental paradigm of the eye-gaze task.

Supplement: Supplementary file 1 — Supplementary file1 (DOCX 110 KB) [file 406_2024_1830_MOESM1_ESM.docx]
