# Supplementary material for: Mentalizing impairments and hypermentalizing bias in individuals with first-episode schizophrenia-spectrum disorder and at-risk mental state: the differential roles of neurocognition and social anxiety
Source: Eur Arch Psychiatry Clin Neurosci. 2024 Jul 3;275(3):907–19. doi: 10.1007/s00406-024-01830-y (PMC11947045; doi:10.1007/s00406-024-01830-y)
Supplement: Supplementary file 2 — Supplementary file2 (DOCX 22 KB) [file 406_2024_1830_MOESM2_ESM.docx]

Supplementary Table 1. Correlations between social cognition and basic demographics, clinical characteristics, and neurocognition in the full sample (*N* = 120).

|  | Comic strip task | Hinting Task | Ambiguous SRGP rate | Unambiguous SRGP rate |
| --- | --- | --- | --- | --- |
| ***Demographic*** |  |  |  |  |
| Age | 0.171 | 0.089 | -0.065 | -0.153 |
| Gender | 0.058 | 0.024 | 0.009 | 0.109 |
| Years of education | **0.399***** | 0.186* | **-0.392***** | **-0.413***** |
| DDD (*N* = 80) | 0.051 | 0.081 | -0.022 | 0.136 |
| ***Clinical characteristics*** |  |  |  |  |
| PANSS positive (*N* = 80) | 0.070 | 0.105 | 0.027 | **0.290**** |
| PANSS negative (*N* = 80) | -0.043 | **-0.295**** | -0.001 | 0.044 |
| PANSS disorganization (*N* = 80) | -0.001 | -0.154 | 0.029 | 0.150 |
| PANSS depression-anxiety (*N* = 80) | 0.199 | 0.042 | 0.119 | 0.134 |
| PANSS excitement/activity (*N* = 80) | -0.034 | -0.092 | -0.023 | 0.072 |
| IRIS item | -0.215* | -0.135 | **0.335***** | **0.390***** |
| LSAS total | **-0.247**** | -0.139 | **0.369***** | **0.410***** |
| PDI total | **-0.317***** | -0.181* | **0.472***** | **0.484***** |
| ***Neurocognition*** |  |  |  |  |
| Executive functions | **0.455***** | **0.258**** | **-0.414***** | **-0.498***** |
| Processing speed | **0.469***** | **0.217*** | **-0.321***** | **-0.410***** |
| Working memory | **0.467***** | **0.280**** | **-0.439***** | **-0.469***** |
| Cognitive composite score | **0.551***** | **0.303***** | **-0.473***** | **-0.548***** |

PANSS: Positive and Negative Syndrome Scale for psychotic symptoms, IRIS: idea of reference interview scale, LSAS: Liebowitz social anxiety scale, PDI: Peter’s delusion inventory, DDD: defined daily dose, DUI: duration of illness, SRGP: self-referential gaze perception. * indicates p<0.05; ** p<0.01; ***p<0.001. Bold indicates significant correlations after controlling for the False Discovery Rate.
